# Drohende Corona-Endemie in einer rheumatologischen Spezialklinik – relative Entwarnung durch konsequente Testung

**DOI:** 10.1007/s00393-021-00959-8

**Published:** 2021-01-25

**Authors:** I. Andreica, B. Guminski, J. Sokolar, A. Patru, X. Baraliakos, J. Braun

**Affiliations:** Rheumazentrum Ruhrgebiet und Ruhr-Universität Bochum, Claudiusstr. 45, 44649 Herne, Deutschland

**Keywords:** Pandemie, COVID-19, Corona-Hygienekonzept, SARS-CoV-2-Antikörpertest, Rheumaspezialklinik, Pandemic, COVID-19, Corona hygiene concept, SARS-CoV-2 antigen test, Hospital for rheumatic diseases

## Abstract

Die SARS-CoV-2-Pandemie hält die meisten Länder der Welt anhaltend in Atem. In Deutschland liegt die Prävalenz von SARS-CoV-2-Infektionen zwar noch unter 2 %, die Zahlen steigen aber seit Wochen auch in Deutschland wieder an. Die rheumatologische Versorgung wurde durch die 1. Welle der Pandemie vorübergehend beeinträchtigt. Wir berichten über die Infektionssituation in der größten deutschen Rheumaspezialklinik, dem Rheumazentrum Ruhrgebiet, weil es hier vor Kurzem während der 2. Welle erstmals zum Auftreten von mehreren SARS-CoV-2-Infektionen an einem Wochenende gekommen ist, was zu einer erheblichen Unruhe bei vielen Beteiligten führte. Die Situation konnte durch konsequentes Testen von Patienten und Mitarbeitern mit dem Antigenschnelltest aufgeklärt und die Lage beruhigt werden. Letztlich waren nur wenige Getestete positiv, und die Verläufe bei den Patienten blieben bis jetzt blande. Dies zeigt auch die Effektivität der seit April konsequent durchgeführten hygienischen Schutzmaßnahmen.

## Abstract (en)

The severe acute respiratory coronavirus type 2 (SARS-CoV-2) pandemic is keeping most countries of the world in suspense. In Germany the prevalence of SARS-CoV‑2 infections is under 2% but for weeks the numbers in Germany have also been increasing. The care in rheumatology was temporarily impaired by the first wave of the pandemic. This article reports the infection situation in the largest specialized rheumatology clinic in Germany, the Rheumatism Center Ruhrgebiet, because recently during the second wave for the first time several SARS-CoV‑2 infections occurred here over one weekend, which led to considerable anxiety in many of those involved. The situation could be clarified by consistent testing of patients and personnel with the rapid antigen test and the situation could be mollified. Ultimately, only a few persons were tested positive and the courses by the patients have so far remained bland. This shows the effectiveness of the protective hygiene measures consistently implemented since April.

Das Rheumazentrum Ruhrgebiet ist das größte deutsche Spezialkrankenhaus für rheumatische Erkrankungen. Im Jahr 2019 wurden ca. 8000 Patienten stationär versorgt, und es gab mehr als 25.000 ambulante Kontakte. Im Jahr 2020 sind bis Anfang November 2020 nur fast 5000 Patienten stationär versorgt worden – wegen der SARS-CoV-2-Pandemie und des initialen Lockdowns im April 2020 ca. 30 % weniger als im Vorjahr. In der allgemeinen spezialfachärztlichen (ASV-)Ambulanz sind es bis Anfang November 2020 wegen der im März und April 2020 vorübergehenden Umstellung auf telefonische Kontakte ca. 10 % weniger als im Vorjahr. Insgesamt war es aber möglich, den stationären und ambulanten Betrieb des Zentrums gut aufrechtzuerhalten, was die tägliche Aufnahmebereitschaft über 24 h und die Notfallversorgung beinhaltet. In unserem Zentrum werden pro Woche durchschnittlich mehr als 10 Patienten notfallmäßig stationär aufgenommen.

Seit Mitte März 2020 wird im Rheumazentrum ein strenges Hygienekonzept verfolgt, und definierte Regeln (Abstand, Händedesinfektion, Mund- und Nasenschutz) werden konsequent eingehalten. Das beinhaltet, dass das gesamte Personal durchgehend einen medizinischen Mund-Nasenschutz und in besonderen Situationen auch „filtering face piece“(FFP)2-Masken trägt. Die Patienten sind verpflichtet, während ihres Aufenthalts im Krankenhaus entweder einen medizinischen Mund-Nasenschutz oder auch Alltagsmasken zu tragen. Dieser Schutz darf nur im eigenen Patientenzimmer abgelegt werden. Ein ausreichender Abstand zu den Bettnachbarn in den Zimmern ist bautechnisch und zum Teil durch neu installierte Plexiglastrennwände gewährt.

In der Zeit vom 01.04.2020 bis 30.10.2020 hatten wir bei den stationären Patienten nur 2 symptomatische Corona-Fälle (<0,1 %). Auch in unserer ASV-Ambulanz gab es, wie kürzlich berichtet, nur wenige Fälle [1].

Am Wochenende vom 23.10. bis 25.10.2020 wurde bei 5 Patienten mit eher mäßigen bzw. keinen Erkältungssymptomen (Zufallsbefund vor Verlegung) SARS-CoV‑2 mittels PCR [10] nachgewiesen. Drei Patienten hatten eine entzündlich rheumatische Erkrankung und erhielten eine immunsupprimierende Therapie. Es wurden unmittelbar entsprechende Schutzmaßnahmen eingeleitet und die Patient*innen entweder isoliert, verlegt oder nach Hause entlassen.

Weil alle 5 Patienten sich an mindestens 2 Tagen zuvor zwar mit Mund-Nasenschutz, aber ansonsten frei im Haus bewegt hatten, wurde beschlossen, im Rahmen einer akuten Bestandsaufnahme alle Patient*innen und Mitarbeiter*innen auf SARS-CoV‑2 zu testen, und zwar mit dem erst vor Kurzem eingeführten Antigenschnelltest von Roche, auch „point of care test“ (POCT) genannt [6].

Dieser Schnelltest hat eine veröffentlicht hohe Sensitivität von 96,52 % und eine sehr gute Spezifität von 99,68 % [6]. Das bedeutet, dass im Idealfall nur 3,48 % aller Getesteten, die infiziert sind, ein (falsch) negatives Testergebnis haben und nur 0,32 % aller Getesteten, die nicht infiziert sind, ein (falsch) positives Testergebnis haben. Die epidemiologischen Grundlagen für den positiven und negativen prädiktiven Wert dieses Tests wurden auf Grundlage der oben genannten Zahlen wie folgt berechnet [9]. Die zugrunde gelegte Gesamtzahl von 20.000 ist letztlich arbiträr und kommt durch die angenommene Prävalenz zustande, um ausreichend große Zahlen (z. B. 1000 Infizierte) für die Kalkulation zu haben.

Geht man von einer sicher (noch) zu hoch geschätzten, nur annähernd realistischen Prävalenz von 5 % der Bevölkerung aus (a + c) / (a + b + c + d), sieht das Bild wie in Tab. 1 dargestellt aus.

**Table 1:** 

	Infiziert +	Infiziert −	Summe
Test positiv	965 (a)	61 (b)	1026 (a + b)
Test negativ	32 (c)	18.942 (d)	18.974 (c + d)
Summe	997 (a + c)	19.003 (b + d)	20.000 (a + b + c + d)

*(a)* Anzahl der richtig positiv getesteten Personen, *(b)* Anzahl der falsch positiv getesteten Personen, *(a + b)* Anzahl aller positiv getesteten Personen, *(c)* Anzahl der falsch negativ getesteten Personen, *(d)* Anzahl der richtig negativ getesteten Personen, *(c + d)* Anzahl aller negativ getesteten Personen

Dabei ist (a) die Anzahl der richtig positiv getesteten Personen = 965 und (a + b) die Anzahl aller positiv getesteten Personen = 1026. Das ergibt einen positiven prädiktiven Wert a / (a + b) von 0,94. Entsprechend ist (d) die Anzahl der richtig negativ getesteten Personen = 18.942 und (c + d) die Anzahl aller negativ getesteten Personen = 18.974. Das ergibt einen negativen prädiktiven Wert d / (c + d) von 0,99. Das bedeutet, dass bei niedriger Prävalenz ein hoher negativer prädiktiver Wert eine Infektion quasi ausschließt.

Es wurden insgesamt 407 Tests innerhalb von 3 Tagen durchgeführt. Insgesamt wurden 201 Mitarbeiter*innen und 206 Patient*innen getestet. Nur 1 Mitarbeiter wollte nicht getestet werden. Alle im Dienst befindlichen Ärzt*innen (*n* = 30) wurden getestet. Es konnten im Mittel etwa 10 Rachenabstriche und Tests pro Stunde durchgeführt werden.

Alle Mitarbeiter*innen wurden negativ getestet. Lediglich 1 Patientin (0,5 % der getesteten Patienten), die klinisch asymptomatisch war, hatte einen positiven Antigenschnelltest. Dieses Ergebnis bestätigte sich anschließend in der Polymerasekettenreaktion(PCR)-Untersuchung. Daraufhin wurden entsprechende Maßnahmen eingeleitet.

Diese Ergebnisse belegen nach unserer Ansicht den Erfolg der konsequenten Masken‑, Abstands- und Hygienestrategie in unserem Spezialkrankenhaus. Nach der anfänglichen Sorge und Unruhe bei Mitarbeiter*innen und Patient*innen, auch in Bezug auf die in der Bevölkerung in den letzten Wochen stetig steigenden COVID-19-Infektionszahlen [5] haben die Ergebnisse des durchgeführten Screenings sehr zur Beruhigung der Situation beigetragen.

Wir finden es auch ermutigend, gelernt zu haben, dass es in bestimmten Situationen möglich und sinnvoll ist, sich z. B. durch die geschilderten Maßnahmen einen relativ schnellen und direkten Überblick über die tatsächliche Infektionslage zu machen.

Nichtsdestoweniger ist beim Testen grundsätzlich ein zielgerichtetes Verfahren wichtig, denn ohne Anlass führt das Testen zu einem falschen Sicherheitsgefühl, weil auch ein negativer SARS-CoV-2-Test eben letztlich nur eine Momentaufnahme darstellt. Darüber hinaus ist wichtig zu betonen, dass die Sensitivität eines Tests grundsätzlich vom Krankheitsstadium, der Viruskonzentration im Nasopharynx und diversen präanalytischen Faktoren wie Qualität und Lokalisation der Abstrichentnahme abhängt. Dementsprechend bleibt abzuwarten, ob die vom Hersteller angegebene Sensitivität im klinischen Alltag ggf. korrigiert werden muss [11].

Seit dem 15.10.2020, gibt es in Deutschland eine nationale Teststrategie [7]. Danach werden in Deutschland auf Grundlage einer durch die behandelnde Ärzt*in bzw. den öffentlichen Gesundheitsdienst getroffenen Entscheidung über die symptomatischen Personen hinaus unter anderem folgende Personengruppen getestet:

- Personen mit für COVID-19 typischen Symptomen – auch bei leichten Symptomen,
- Kontaktpersonen: Personen, die Kontakt zu einer mit SARS-CoV‑2 infizierten Person hatten, z. B. Mitglieder desselben Haushalts oder Personen, die über die Corona-Warn-App als Kontaktpersonen identifiziert wurden,
- Gemeinschaftseinrichtungen: Personen in Gemeinschaftseinrichtungen und -unterkünften (z. B. Arztpraxen, Schulen, Kitas, in Geflüchteten- und Notunterkünften sowie in Justizvollzugsanstalten), wenn in der Einrichtung eine infizierte Person ermittelt wurde,
- Bewohnerinnen und Bewohner von Betreuungseinrichtungen sowie Patientinnen und Patienten: Patienten und Bewohner vor (Wieder‑)Aufnahme und bei einem SARS-CoV-2-Ausbruch in patientennahen Einrichtungen wie Krankenhäusern, stationären Pflegeeinrichtungen, Behinderteneinrichtungen, Reha-Einrichtungen und der ambulanten Pflege,
- Personal in patientennahen Einrichtungen: stichprobenartig unabhängig von Fällen,
- Besucherinnen und Besucher von patientennahen Einrichtungen: vor Besuch der Einrichtung.
- in Regionen mit vielen Neuinfektionen (mindestens 50 Fälle pro 100.000 Einwohner über 7 Tage) können Teile der Bevölkerung bzw. die gesamte Bevölkerung stichprobenartig getestet werden.

Die besonderen Regelungen für Reisende wurden bei dieser Aufzählung nicht berücksichtigt. Die genannten Personengruppen sollen entweder mit einem PCR- oder einem Antigentest getestet werden [7].

Durch hohe Fallzahlen gekennzeichnete aktuelle Pandemiesituation (Stand 22.01.2021) mit den neu aufgetretenen offensichtlich infektiöseren Mutationen haben sich verschiedene Regelungen geändert. Deshalb können wir an dieser Stelle nur darauf hinweisen, dass der Artikel Anfang November geschrieben wurde und sich die gemachten Angaben auf den damals aktuellen Zeitraum beziehen.

In der Stadt Herne wurde seit Beginn der Pandemie bei 1711 Personen eine Infektion mit COVID-19 nachgewiesen. Aktuell (Stand 03.11.2020) sind hier 614 Personen infiziert, von denen 46 stationär in anderen Krankenhäusern behandelt werden [3]. Da nur < 20 % unserer Patienten aus Herne kommen, sind die Zahlen für Nordrhein-Westfalen (NRW) wichtiger: Hier haben sich seit dem Ausbruch der Epidemie insgesamt 153.766 Menschen mit SARS-CoV‑2 infiziert (0,89 % der Bevölkerung). In dem Zusammenhang wurden 2267 Todesfälle (1,47 % aller Infizierten) erfasst [4].

In der Woche nach dem Screening wurden im Rheumazentrum Ruhrgebiet vor der stationären Aufnahme insgesamt 131 Patient*innen mit dem Antigenschnelltest getestet – es waren alle negativ. Alle 9 in unserem Zentrum bislang positiv getesteten Patient*innen hatten einen relativ blanden bzw. asymptomatischen Verlauf, eine intensivmedizinische Behandlung war in keinem Fall erforderlich. Das sind kleine Fallzahlen, die aber sowohl unsere bisherigen Erfahrungen [1] und die anderer europäischer Rheumatologen [8] bestätigen. Nichtsdestoweniger gibt es auch andere Berichte wie zuletzt aus den USA [2], dass bestimmte Bevölkerungsgruppen, wie z. B. ethnische Minderheiten mit rheumatischen Erkrankungen, in wahrscheinlichem Zusammenhang mit der gesamten Organisation des nationalen Gesundheitssystems durchaus problematischere Outcomes haben können.

## References

[1] Andreica I, Kiefer D, Rezniczek GA (2020). Comment on ‘Characteristics associated with hospitalisation for COVID-19 in people with rheumatic disease: data from the COVID-19 global rheumatology alliance physician-reported registry’ by Gianfrancesco M et al. Ann Rheum Dis.

[2] Gianfrancesco MA, Leykina LA, Izadi Z et al (2020) Race/ethnicity association with COVID-19 outcomes in rheumatic disease: Data from the COVID-19 Global Rheumatology Alliance Physician Registry. Arthritis Rheumatol. 10.1002/art.41567 10.1002/art.41567 PMC8467766 33146001

[3] http://www.herne.de/Stadt-und-Leben/Gesundheit/Informationen-zum-Coronavirus. Zugegriffen: 3. Nov. 2020

[4] http://www.mags.nrw/coronavirus-fallzahlen-nrw. Zugegriffen: 3. Nov. 2020

[5] http://www.rki.de/SharedDocs/Newsletter/Infektionsschutz/2020/201027-NewsletterInfektionsschutz. Zugegriffen: 3. Nov. 2020

[6] http://www.roche.de/res/content/11722/packungsbeilage_sars-cov-2_rapid_antigen_test.pdf. Zugegriffen: 3. Nov. 2020

[7] http://www.zusammengegencorona.de/informieren/informationen-zum-test/#faqitem=4f34d545-7b49-5976-8b43-b7c80d68da47. Zugegriffen: 3. Nov. 2020

[8] Sarzi-Puttini P, Marotto D, Caporali R (2020). Prevalence of COVID infections in a population of rheumatic patients from Lombardy and Marche treated with biological drugs or small molecules: a multicentre retrospective study. J Autoimmun.

[9] Schlenger R (2020) Antigentests auf SARS-CoV-2: Der Preis der Schnelligkeit. Dtsch Ärztebl 117(44):A-2101/B-1787

[10] Vogels CBF, Brito AF, Wyllie AL (2020). Analytical sensitivity and efficiency comparisons of SARS-CoV-2 RT-qPCR primer-probe sets. Nat Microbiol.

[11] Gillissen A (2020). Übersicht zu Sensitivität und Spezifität des SARS-CoV-2-Nachweises mittels PCR. Pneumo News.

